# The Role of Nutrition, Oxidative Stress, and Trace Elements in the Pathophysiology of Autism Spectrum Disorders

**DOI:** 10.3390/ijms26020808

**Published:** 2025-01-18

**Authors:** Anna Długosz, Marcin Wróblewski, Błażej Błaszak, Joanna Szulc

**Affiliations:** 1 Department of Food Industry Technology and Engineering, Faculty of Chemical Technology and Engineering, Bydgoszcz University of Science and Technology, 3 Seminaryjna St., 85-326 Bydgoszcz, Poland; blazej.blaszak@pbs.edu.pl (B.B.); joanna.szulc@pbs.edu.pl (J.S.); 2 Department of Medical Biology and Biochemistry, Faculty of Medicine, Ludwik Rydygier Collegium Medicum in Bydgoszcz, Nicolaus Copernicus University in Toruń, 24 Karłowicza St., 85-092 Bydgoszcz, Poland; marcin.wroblewski@cm.umk.pl

**Keywords:** autism spectrum disorder (ASD), gluten-free and casein-free (GFCF) diet, ketogenic diet (KD), nutritional interventions, oxidative stress, trace elements

## Abstract

Autism spectrum disorder (ASD) is a neurodevelopmental condition characterized by deficits in social communication and interaction, alongside repetitive behaviors, and atypical sensory-motor patterns. The growing prevalence of ASD has driven substantial advancements in research aimed at understanding its etiology, preventing its onset, and mitigating its impact. This ongoing effort necessitates continuous updates to the body of knowledge and the identification of previously unexplored factors. The present study addresses this need by examining the roles of nutrition, oxidative stress, and trace elements in the pathophysiology of ASD. In this review, an overview is provided of the key dietary recommendations for individuals with ASD, including gluten-free and casein-free (GFCF) diets, ketogenic diets (KDs), and other nutritional interventions. Furthermore, it explores the involvement of oxidative stress in ASD and highlights the significance of trace elements in maintaining neuropsychiatric health. The impact of these factors on molecular and cellular mechanisms was discussed, alongside therapeutic strategies and their efficacy in managing ASD.

## 1. Introduction

Autism spectrum disorder (ASD) is classified as a neurodevelopmental disorder characterized by deficits in social communication and interaction, as well as repetitive (stereotypic) and atypical sensory–motor behaviors. These challenges can significantly impact individuals with ASD, leading to difficulties in social interactions and reduced employment opportunities [1,2,3]. ASD also imposes a substantial economic burden, primarily due to the support required by adults who are unable to live independently. This includes increased healthcare and educational expenses, as well as loss of income for caregivers. While ASD is a biological condition, its primary treatments involve educational and behavioral interventions, with medications serving as an adjunct [3].

The symptomatology of ASD is highly heterogeneous, with significant variability among individuals. According to the fifth edition of the Diagnostic and Statistical Manual of Mental Disorders, fifth edition (DSM-5), ASD is defined by impairments in the two core domains of social communication and repetitive behaviors. For an ASD diagnosis, individuals must exhibit at least three symptoms in the social communication domain and at least two in the repetitive behavior domain [4]. Additionally, the American Psychiatric Association emphasizes the importance of screening for and ruling out other medical conditions, such as attention deficit hyperactivity disorder (ADHD), anxiety, depression, and gastrointestinal (GI) issues, to ensure an accurate diagnosis [2,3].

The prevalence of ASD has risen significantly in recent years. In 2006, the estimated diagnosis rate was 1 in 150 children; this has since increased to 1 in 36 children [2,5]. This prevalence is believed to be consistent across racial, ethnic, and socioeconomic groups. However, gender disparities are evident, with ASD occurring four to five times more frequently in boys than in girls. Prevalence within families varies depending on the biological relationship among siblings, being highest among identical twins and lower among non-twin siblings. This risk can increase further if one sibling is already affected.

The etiology of ASD remains poorly understood, though it is widely accepted to involve a combination of genetic, environmental, and other factors. Contributing factors include genetic and chromosomal abnormalities, parental psychiatric disorders, advanced parental age, premature birth, low birth weight, and maternal exposure to insecticides, infections, or psychotropic medications during pregnancy [1,2,3,5]. Maternal nutrition during pregnancy also plays a critical role in ASD development. Moreover, studies have confirmed that the nutritional status of children with ASD significantly differs from that of typically developing children [6,7]. Nutritional imbalances, including deficiencies and excesses of specific nutrients, are linked to dietary behaviors such as food aversion, habitual eating patterns, and food selectivity. These nutritional deficits can adversely affect children physically and emotionally, potentially exacerbating autistic symptoms [6,8].

Numerous studies are being conducted on the impact of nutritional status and associated dietary interventions, trace elements, and oxidative stress on the pathophysiology of ASD and the condition of individuals affected by ASD [9]. Simultaneously, this necessitates the continuous updating of knowledge and the analysis of yet-to-be-discovered factors. The aim of this paper was to explore the roles of nutrition, oxidative stress, and trace elements in the pathophysiology of autism spectrum disorder (ASD). Therefore, in this paper, the roles of nutrition, oxidative stress, and trace elements in the pathophysiology of ASD were explored. It provides an overview of key dietary recommendations for individuals with ASD, including gluten-free and casein-free (GFCF) diet, ketogenic diet (KD), and other nutritional interventions. Furthermore, it examines the involvement of oxidative stress in ASD and underscores the importance of trace elements in neuropsychiatric health. The influence of these factors on molecular and cellular mechanisms is discussed, along with therapeutic strategies and their efficacy in managing ASD.

## 2. Methods

**Figure 1:**
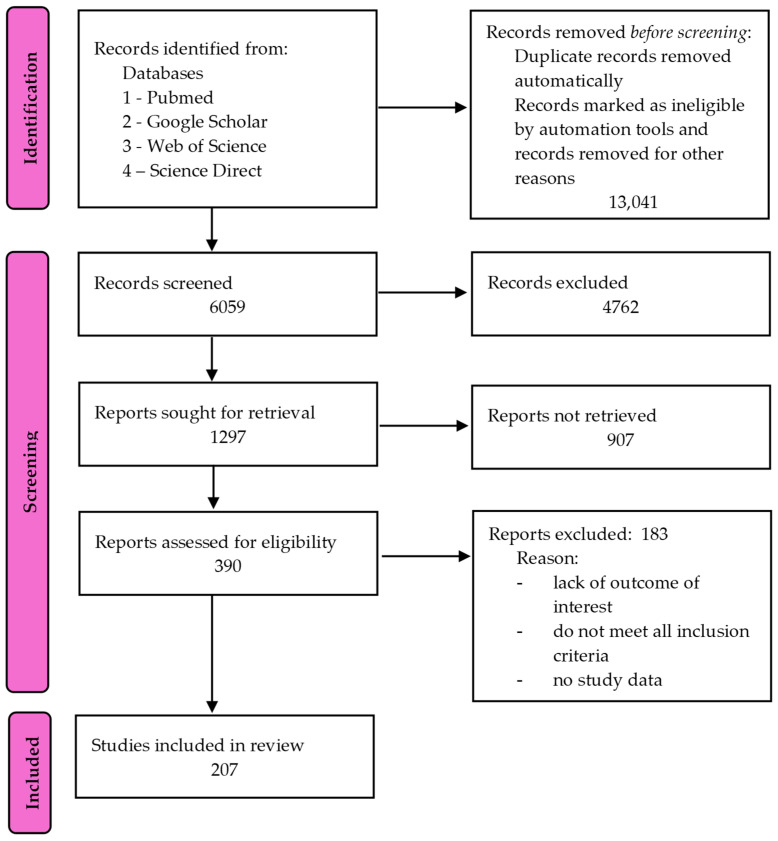
Literature search strategy.

We conducted an extensive literature search using the ISI Web of Science/PubMed/Science Direct/Google Scholar database containing information on the impact of diet, nutrition, oxidative stress, and trace elements on the pathophysiology of ASD. The literature was from the period from March 2024 to December 2024. The following keywords were used in the data search: (“nutrition” and “ASD”, “diet”); (“oxidative stress” and “ASD”); (“oxidative stress” and “selenium”, “zinc”, “copper”, “selenium”, “iron”, “magnesium”); (“reactive oxygen species” and “ASD”); (“trace elements” and “ASD”, “oxidative stress”, “ROS”). There were no restrictions in collecting the data. No language restrictions were applied during the analysis. Rather, we tried to select articles from the last 20 years. After searching, we further examined the full text of the literature to determine eligibility for inclusion in this review. Editorials, conference abstracts, and studies with incomplete or unavailable data were excluded. Articles that did not contain data on the influence of the KD, GFCF diet, probiotics, prebiotics, omega-3 fatty acids, microbiome, oxidative stress, trace elements such as iron, selenium, zinc, copper, magnesium on the etiology and course of ASD were excluded. The discussed methodology allowed for the selection of records on which this review was based (Figure 1).

## 3. Nutrition and ASD

Nutritional interventions play a key role in managing ASD. This review presents the clinical evidence for the main dietary approaches, including the GFCF, the KD, and other interventions such as probiotics, prebiotics, omega-3 fatty acid supplementation, and the gut microbiome. The effectiveness of these strategies and their potential impact on ASD symptoms will be discussed to guide future therapeutic approaches.

### 3.1. GFCF Diet in ASD

The GFCF diet is a dietary intervention commonly explored in the management of ASD. The premise of this diet is based on the hypothesis that gluten (found in wheat, rye, and barley) and casein (a protein in milk and dairy products) may contribute to ASD symptoms through mechanisms involving the gut–brain axis, immune dysregulation, and opioid-like effects. While the GFCF diet has gained popularity among families of individuals with ASD, clinical evidence supporting its effectiveness remains mixed, necessitating further investigation [10,11,12,13,14,15].

Gluten and casein are believed to break down into peptide fragments, such as gliadorphins and casomorphins, that may mimic opioid-like activity [16]. These peptides could cross a “leaky” intestinal barrier, enter systemic circulation, and potentially influence brain function, exacerbating ASD symptoms such as hyperactivity, irritability, and communication deficits. Many individuals with ASD exhibit GI dysfunctions, including increased intestinal permeability (“leaky gut”) and gut microbiota dysbiosis [17]. Gluten and casein are thought to exacerbate these issues, leading to systemic inflammation and altered neurodevelopment through immune-mediated pathways [13,16,18,19].

Food-derived antigens such as gluten and casein may provoke abnormal immune responses in susceptible individuals. Increased levels of antibodies to gluten and casein have been observed in some individuals with ASD, suggesting immune involvement in their pathology [10].

The randomized controlled trial (RCT) by Hyman et al. [20] evaluated the effects of a GFCF diet in children with ASD over 24 weeks. The study found no significant differences in core ASD symptoms, such as communication and socialization, between the intervention and control groups. However, anecdotal reports from the parents suggested improvements in sleep and GI issues for some children [20]. Baspinar and Yardimci [13] conducted a study assessing GI outcomes in children with ASD following a GFCF diet. Findings revealed a reduction in GI symptoms such as diarrhea, constipation, and abdominal pain, which were associated with improved behavior and attention. A study by González-Domenech et al. [21] examined children with ASD on a GFCF diet and found no correlation between urinary beta-casomorphin levels and behavioral improvements. This raises questions about the role of opioid peptides in ASD pathology. Recent research has highlighted that the effectiveness of the GFCF diet may depend on individual factors, such as pre-existing GI symptoms, biomarkers of immune sensitivity, or genetic predispositions [12,13]. Children with significant GI issues or elevated anti-gluten antibodies appear to benefit most from the diet.

The GFCF diet requires the complete elimination of gluten and casein, which can be challenging to maintain, particularly for individuals with ASD who may already have restricted eating habits or food selectivity. Excluding gluten and dairy products may lead to deficiencies in essential nutrients such as calcium, vitamin D, and certain B vitamins. Careful dietary planning and supplementation are necessary to mitigate these risks [22,23]. Placebo-controlled studies have demonstrated that parental expectations and biases can significantly influence perceived improvements, complicating the interpretation of outcomes in GFCF dietary trials [20].

The implementation of a GFCF diet should be guided by healthcare professionals, including dietitians, to ensure nutritional adequacy and monitor for potential adverse effects. Not all individuals with ASD will respond to the GFCF diet. Identifying candidates with specific markers, such as GI dysfunction, elevated gluten or casein antibodies, or food allergies, can improve the likelihood of success. A trial period of 3–6 months is recommended to evaluate the diet’s impact on ASD symptoms. Careful tracking of behavioral, GI, and developmental outcomes during this period is crucial. Adequate intake of calcium, vitamin D, and other key nutrients should be ensured through fortified foods or supplements to prevent deficiencies related to dietary restrictions [10,13].

The GFCF diet remains a widely debated intervention in ASD management. To strengthen the evidence base, future studies should focus on the following:

- conducting large, well-controlled RCTs with standardized protocols to evaluate its efficacy across different ASD subpopulations;
- exploring biomarkers, such as gut microbiota composition, inflammatory markers, and genetic polymorphisms, to identify responders versus non-responders;
- investigating the long-term safety and sustainability of the GFCF diet in children and adults with ASD.

A better understanding of the diet’s mechanisms and identification of suitable candidates will help optimize its use as part of a personalized approach to ASD management.

### 3.2. KD in ASD

The KD—a high-fat, low-carbohydrate, and moderate-protein dietary regimen—is widely recognized for its efficacy in managing refractory epilepsy. Its application in ASD has garnered significant interest due to overlapping pathophysiological mechanisms, including mitochondrial dysfunction, neuroinflammation, oxidative stress, and altered neurotransmitter activity. Emerging evidence suggests that KD may hold therapeutic potential for certain individuals with ASD, particularly those with comorbid epilepsy or metabolic dysfunctions [24,25].

The KD promotes the production of ketone bodies, including β-hydroxybutyrate and acetoacetate, which serve as alternative energy sources for the brain. These ketones modulate the balance between gamma-aminobutyric acid (GABA) and glutamate, enhancing inhibitory neurotransmission and potentially reducing the neural hyperexcitability associated with ASD symptoms [24,26,27,28,29]. This mechanism is particularly relevant for individuals with the heightened excitatory/inhibitory imbalances observed in ASD. Mitochondrial dysfunction is frequently observed in ASD and is characterized by impaired energy production and heightened oxidative stress. The KD supports mitochondrial biogenesis and function by improving adenosine triphosphate (ATP) production and reducing the accumulation of reactive oxygen species (ROS). These effects mitigate cellular stress and contribute to neural resilience [24,30]. Chronic neuroinflammation is a key component of ASD pathology. The KD reduces levels of pro-inflammatory cytokines, including tumor necrosis factor-alpha (TNF-α) and interleukin-6 (IL-6), while increasing anti-inflammatory markers. This anti-inflammatory shift may help restore homeostasis in the central nervous system and improve cognitive and behavioral outcomes. In the study of Olivito et al. [31], the impact of the KD on ASD-like behaviors in BTBR T+ Itpr3tf/J mice was assessed. It was found that the KD alleviates these behaviors by reducing inflammatory factors and remodeling the gut microbiota [31]. Other studies have suggested that the KD may influence the gut microbiome, which in turn could modify the inflammatory response and improve cognitive functions in individuals with ASD [32]. ASD is often associated with dysbiosis of the gut microbiota, which can exacerbate behavioral and GI symptoms. KD positively alters gut microbiota composition, increasing beneficial bacteria such as *Akkermansia* and *Bifidobacterium*, which are associated with improved gut integrity, reduced systemic inflammation, and enhanced behavior [32,33,34].

The study on children with ASD using a modified ketogenic gluten-free diet with medium-chain triglycerides (MCTs) showed significant improvements in social skills and communication, as measured by the Autism Diagnostic Observation Schedule, 2nd edition (ADOS-2), particularly in social affect scores. These improvements were sustained over six months. However, no significant changes were observed in restricted and repetitive behaviors. Further research is needed to explore the diet’s impact on these domains [35]. Evangeliou et al. [36] performed a pilot study on 30 children with ASD who adhered to a KD. Approximately 60% of participants demonstrated reductions in irritability and hyperactivity, with pronounced benefits observed in individuals with concurrent epilepsy [36]. The pilot study by Mu et al. [37], which investigated behavioral parameters in children with ASD following a 3-month treatment with a modified KD, identified associations between plasma metabolites, trace elements, and behavioral outcomes, suggesting the potential benefits of the KD in improving key symptoms of ASD. Studies by Li et al. [24] and Allan et al. [32] highlighted the benefits of KD in alleviating GI symptoms commonly associated with ASD, such as constipation and bloating. Improvement in these symptoms was associated with better behavioral regulation, emphasizing the importance of addressing gut health in the treatment of ASD.

While the KD shows promise, its implementation presents several challenges. The strict macronutrient requirements of the KD can be difficult to maintain, particularly for individuals with ASD who often exhibit sensory sensitivities or selective eating habits. Without proper planning, KD can lead to deficiencies in essential nutrients, including fat-soluble vitamins (A, D, E, K) and minerals such as calcium and magnesium. Common side effects include GI discomfort, hyperlipidemia, and, in rare cases, more severe complications such as kidney stones or pancreatitis. Careful monitoring is essential to mitigate these risks [24].

The KD should only be initiated under the guidance of a multidisciplinary team, including pediatricians, neurologists, and dietitians. Regular monitoring of metabolic parameters, nutritional status, and potential adverse effects is essential. Not all individuals with ASD will benefit equally from KD. Biomarkers such as mitochondrial function, inflammatory profiles, and gut microbiota composition may help identify individuals most likely to respond favorably. The current evidence suggests that a trial period of 3–6 months is appropriate to evaluate efficacy. Long-term sustainability should be reassessed periodically, balancing benefits against potential risks and dietary burdens [24,38,39].

The emerging evidence suggests potential benefits of the KD in managing ASD symptoms, but further studies are essential to establish its efficacy and refine its application.

- Large-scale, placebo-controlled studies are needed to confirm the effectiveness of KD in ASD treatment.
- The influence of KD on ketone metabolism, synaptic plasticity, and gut–brain communication requires deeper exploration.
- Genetic, metabolic, and microbiome profiles should be analyzed to tailor the KD for specific ASD subgroups.
- The potential risks, such as nutrient deficiencies and GI issues, need thorough assessment.
- The impact of a KD on gut microbiota and its relationship to ASD symptoms warrants further investigation.
- The KD should be studied alongside behavioral or pharmacological treatments to determine synergistic effects.
- Less restrictive versions of the KD should be created to improve adherence and accessibility.

Further research into these areas could help optimize the KD as a viable and personalized treatment option for individuals with ASD.

### 3.3. Probiotics and Prebiotics in ASD

Probiotics and prebiotics have garnered increasing interest as dietary interventions for ASD, given the critical role of the gut–brain axis in ASD pathophysiology. The GI symptoms, such as constipation, diarrhea, and abdominal pain, are prevalent in individuals with ASD, and evidence suggests that gut microbiota dysbiosis may exacerbate both GI and behavioral symptoms. Probiotics, live microorganisms conferring health benefits, and prebiotics, dietary fibers that promote the growth of beneficial gut bacteria, aim to restore microbiota balance and improve associated symptoms in ASD [40,41,42].

Individuals with ASD often exhibit altered gut microbiota profiles, characterized by reduced diversity and imbalances in key bacterial species such as *Bacteroides*, *Clostridium*, and *Firmicutes*. Probiotics and prebiotics can help restore microbial balance by increasing beneficial bacteria like *Lactobacillus* and *Bifidobacterium* while reducing pathogenic species [17,43,44,45,46]. Increased intestinal permeability is frequently reported in ASD. Probiotics strengthen tight junctions in the intestinal lining, reducing systemic inflammation and the translocation of bacterial endotoxins such as lipopolysaccharides (LPSs), which are implicated in neuroinflammation [47,48]. Probiotics modulate the immune system by reducing pro-inflammatory cytokines, such as TNF-α and IL-6, and increasing anti-inflammatory cytokines like interleukin-10 (IL-10). This may alleviate the neuroinflammation associated with ASD [41,49,50]. Certain gut bacteria produce neurotransmitters such as GABA, serotonin, and dopamine, which influence brain function. Probiotic strains like *Lactobacillus rhamnosus* have been shown to enhance GABAergic signaling, potentially ameliorating anxiety and behavioral symptoms in ASD [41,51].

A study by Grimaldi et al. [52] found that a synbiotic (combination of probiotics and prebiotics) significantly improved GI symptoms such as constipation and diarrhea in children with ASD. These improvements were associated with changes in the gut microbiota composition, including increased levels of *Bifidobacterium* and *Prevotella*. Kang et al. [53] investigated fecal microbiota transplantation (FMT) as an adjunct to probiotics in children with ASD and observed not only GI symptom relief but also significant improvements in ASD behavioral symptoms, including reduced irritability and better social communication, as measured by the Childhood Autism Rating Scale (CARS). Parracho et al. [54] conducted a placebo-controlled trial with a probiotic supplement containing *Lactobacillus plantarum* and reported reductions in anxiety and hyperactivity, suggesting a potential role in modulating behavioral symptoms.

Not all probiotics are equally effective. Different bacterial strains may produce varying effects, and selecting the right strain is critical. For instance, *Lactobacillus reuteri* has been linked to improved social behavior in preclinical models, while *Bifidobacterium longum* is associated with reduced anxiety and GI symptoms [55]. Probiotics and prebiotics are generally well-tolerated, but adherence can be challenging in children with ASD, particularly those with sensory sensitivities or aversions to specific tastes or textures. Not all individuals with ASD respond to probiotics and prebiotics. Factors such as baseline microbiota composition, severity of GI symptoms, and age may influence the effectiveness of these interventions [53].

Recent research has highlighted the significant influence of the gut microbiome on the activation of the aryl hydrocarbon receptor (AhR), a ligand-activated transcription factor involved in mediating the effects of environmental pollutants and dietary components. The gut microbiota produces metabolites that can modulate AhR activity, influencing processes such as immune tolerance, intestinal homeostasis, and barrier integrity [56]. This interaction between the microbiome and AhR is crucial in the gut–brain axis, where disruptions in AhR signaling have been linked to various neurological disorders. Alterations in AhR activity, influenced by microbiome-derived metabolites, can impact neuroinflammation and neuroplasticity, processes associated with mental health conditions like depression and anxiety [57]. Understanding the microbiome–AhR–brain axis offers potential therapeutic avenues for these disorders, providing insights into novel approaches for prevention and treatment.

Probiotic and prebiotic supplementation should be tailored to individual needs, with consideration of baseline gut microbiota profiles and specific symptoms such as GI dysfunction or anxiety. Probiotic strains such as *Lactobacillus rhamnosus*, *Bifidobacterium bifidum*, and *Lactobacillus reuteri* have demonstrated potential benefits in ASD and should be prioritized. A trial period of at least 8–12 weeks is recommended to assess efficacy. Careful monitoring of GI, behavioral, and cognitive outcomes during this period is crucial. Probiotics and prebiotics should be used as part of a comprehensive treatment plan, alongside behavioral therapies, dietary modifications, and other medical interventions [46,52,54].

Despite promising findings, the evidence base for probiotics and prebiotics in ASD remains limited by small sample sizes, heterogeneity in study designs, and variability in outcomes. Future research should focus on the following:

- conducting large-scale, placebo-controlled RCTs to establish the efficacy of specific strains and formulations;
- exploring the interplay between gut microbiota, immune modulation, and brain function in ASD;
- Investigating personalized microbiome-based interventions, such as targeted prebiotics and FMT.

### 3.4. Omega-3 Fatty Acids in ASD

Omega-3 fatty acids, particularly eicosapentaenoic acid (EPA) and docosahexaenoic acid (DHA), are essential polyunsaturated fatty acids (PUFAs) with known roles in neurodevelopment, synaptic plasticity, and anti-inflammatory processes. Given the neuroinflammatory and neurotransmitter imbalances observed in ASD, omega-3 supplementation has been explored as a potential intervention [58,59,60,61].

Omega-3 fatty acids reduce levels of pro-inflammatory cytokines, such as IL-6 and TNF-α, while promoting the production of anti-inflammatory molecules like resolvins and protectins [62,63]. DHA is a critical component of neuronal membranes, enhancing fluidity and neurotransmission. It is essential for the functioning of key receptors such as the *N*-methyl-*D*-aspartate receptor (NMDA), which are implicated in ASD-related learning and memory deficits [64,65]. Omega-3 fatty acids influence the synthesis, release, and reuptake of dopamine and serotonin, potentially mitigating the mood dysregulation and anxiety often seen in ASD [66]. During neurodevelopment, omega-3 fatty acids contribute to neuronal migration, axonal growth, and myelination, processes that may be disrupted in ASD [67,68,69].

Amminger et al. [70] conducted a double-blind, randomized, placebo-controlled pilot study and found that omega-3 fatty acids may serve as an effective treatment for children with autism, particularly in reducing hyperactivity and stereotypy. Similarly, de Andrade Wobido et al. [71] presented a meta-analysis demonstrating that omega-3 supplementation could improve ASD symptoms, including reductions in anxiety and enhancements in social communication. Further supporting these findings, Cheng et al. [72] conducted a clinical trial that concluded omega-3 supplementation may lead to improvements in hyperactivity, lethargy, and stereotypy among individuals with ASD. Collectively, these studies have suggested that omega-3 supplementation holds promise in managing certain behavioral symptoms of ASD. However, the variability in responses emphasizes the necessity for further research to establish optimal dosages, treatment durations, and to identify the individuals most likely to benefit from these interventions.

Recent studies have shown that omega-3 PUFAs, such as EPA and DHA, can activate the peroxisome proliferator-activated receptor gamma (PPARγ). This activation plays a crucial role in reducing inflammation and regulating genes related to neuronal survival and synaptic plasticity, processes critical to mental health. The activation of PPARγ by omega-3 PUFAs has been linked to improvements in mood regulation and reduction in depressive symptoms. The research suggests that omega-3 PUFAs may offer therapeutic potential for mental health conditions through this mechanism [73,74].

The heterogeneity of ASD contributes to differing responses to omega-3 supplementation, making it essential to identify the subgroups that are most likely to benefit. The ideal EPA/DHA ratio and dosage remain unclear, with studies using doses ranging from 300 mg to 2000 mg per day. Omega-3 supplements are generally well-tolerated, though GI discomfort or a fishy aftertaste may reduce compliance in some individuals [75,76].

Emerging evidence underscores the potential of omega-3 supplementation in ASD, but further investigation is essential to clarify its role and optimize its application.

- Larger, placebo-controlled trials are needed to determine the efficacy of omega-3 supplementation in ASD across diverse populations.
- Biomarker studies, including analyses of baseline omega-3 levels and inflammatory markers, could help identify the individuals most likely to benefit.
- Research should examine the effects of omega-3 supplementation in conjunction with behavioral or pharmacological interventions.
- Studies with extended follow-up periods are necessary to assess the durability of clinical improvements.

Trials investigating the ideal dosage and EPA/DHA ratio tailored to ASD are critical for maximizing efficacy.

Omega-3 fatty acids show promise as a safe and accessible intervention for addressing certain symptoms of ASD. With further high-quality research, supplementation could become a valuable component of personalized treatment strategies.

### 3.5. Gut Microbiome in ASD

The gut microbiome, which consists of trillions of microorganisms residing in the GI tract, plays a crucial role in human health, influencing immune function, metabolism, and even brain activity. In recent years, the gut–brain axis has become an area of significant interest in ASD. Dysbiosis, an imbalance in gut microbiota composition, has been increasingly recognized as a potential factor contributing to ASD symptoms. The emerging evidence suggests that alterations in the gut microbiome may influence neurodevelopmental processes, neuroinflammation, and behavioral outcomes in individuals with ASD [44,77,78].

The gut and brain communicate through the gut–brain axis, involving neural, hormonal, and immune pathways. Microbial metabolites such as short-chain fatty acids (SCFAs), produced by gut bacteria from dietary fibers, can influence brain function by modulating inflammation and neurotransmitter synthesis [79,80]. Dysbiosis in the gut microbiome can lead to an overactive immune response, increasing systemic inflammation. This chronic inflammation is believed to affect brain development and function, contributing to ASD-related behaviors [17,50,81,82]. Inflammatory cytokines produced in the gut can enter the bloodstream and impact the central nervous system. Certain gut bacteria are involved in the synthesis of neurotransmitters such as serotonin, which plays a crucial role in regulating mood, behavior, and social interaction. Altered gut microbiota composition in individuals with ASD may lead to imbalances in neurotransmitter levels, potentially contributing to social and communication difficulties [83,84,85]. SCFAs like butyrate, propionate, and acetate, which are produced by gut bacteria, have been shown to impact the development of the blood–brain barrier, promote neurogenesis, and reduce neuroinflammation. These metabolites may help modulate brain function and improve symptoms in ASD [79,86].

Studies have consistently found differences in the gut microbiota composition of individuals with ASD compared to neurotypical individuals. For example, lower diversity in the gut microbiota and an overgrowth of pathogenic bacteria such as *Clostridia* have been reported in children with ASD [87]. Clinical studies have shown that interventions aimed at modifying the gut microbiome, such as probiotic supplementation or dietary changes, may lead to improvements in ASD symptoms [88,89]. A study by Kang et al. [53] demonstrated that children with ASD who received probiotics for eight weeks showed significant improvements in social behaviors and communication skills. GI issues, such as constipation, diarrhea, and bloating, are commonly reported in individuals with ASD. These symptoms may further exacerbate behavioral challenges. Treatment aimed at balancing the gut microbiome may help alleviate these GI symptoms and, in turn, improve behavioral outcomes [90]. In some studies, FMT has shown promise in treating ASD-related symptoms by restoring a balanced gut microbiome. Though still experimental, FMT may offer a novel approach to improving both GI and behavioral symptoms in individuals with ASD [53,91,92].

The human microbiome is highly individualized, and the diversity of gut bacteria varies significantly from person to person. This variability complicates the identification of specific microbial signatures associated with ASD. While promising, many interventions aimed at altering the gut microbiome, such as probiotics or dietary changes, are still in the early stages of research. Long-term studies with larger sample sizes are needed to confirm their effectiveness. Some treatments, such as FMT, carry potential risks, including infections or adverse reactions. These therapies should be carefully considered and only administered under medical supervision [83,91,92].

- Future research should focus on identifying specific microbial communities or metabolites that are most beneficial for individuals with ASD. Personalized treatments based on microbiome profiles may maximize therapeutic outcomes.
- Long-term studies examining the effects of microbiome modulation on ASD symptoms are crucial for determining the sustainability and safety of these interventions.
- Further research into how the gut microbiome influences brain development and neuroplasticity is needed. Understanding the underlying mechanisms of gut–brain communication will provide insights into potential therapeutic strategies.
- Combining microbiome-targeted interventions with other treatments, such as behavioral therapy or pharmacological approaches, may lead to more comprehensive and effective ASD management strategies.

The gut microbiome plays a crucial role in the pathophysiology of ASD, influencing behavior, immune function, and brain development. While promising evidence supports the therapeutic potential of microbiome modulation, further high-quality, large-scale studies are necessary to validate these findings. Personalized approaches targeting the gut microbiome, in conjunction with other interventions, could offer a novel strategy for managing ASD symptoms and improving quality of life for affected individuals.

In conclusion, while the current research on dietary interventions in ASD, including the GFCF diet and KD, has yielded promising results, further high-quality studies are necessary to fully understand their efficacy and mechanisms of action. Additionally, interventions targeting the gut microbiome, supplementation with omega-3 fatty acids, and the use of probiotics and prebiotics offer potential avenues for personalized treatment approaches. A more refined understanding of the interactions between diet, neurobiology, and GI function in individuals with ASD will help guide the development of effective, individualized nutritional therapies.

Recent research has highlighted the importance of addressing nutritional deficiencies in children with ASD, particularly through targeted interventions. Studies have shown that deficiencies in essential vitamins, such as vitamin D and vitamin A, are commonly observed in individuals with ASD and may contribute to the exacerbation of symptoms. For example, Dong et al. [93] found a correlation between serum menaquinone-4 concentrations and developmental outcomes in children with ASD, suggesting that vitamin K may also play a role in neurodevelopment. Similarly, Dong et al. [94] demonstrated a link between low serum levels of 25-hydroxyvitamin D and core symptoms of ASD, emphasizing the importance of maintaining adequate vitamin D levels for improving developmental outcomes. Moreover, Zhang et al. [95] explored the relationship between plasma vitamin levels and ASD, revealing how deficiencies in certain vitamins could affect brain function and behavior. These findings highlight the need for interventions aimed at correcting these nutritional deficiencies to potentially improve both gut health and behavioral symptoms in ASD. Additionally, Kacimi et al. [96] reviewed the connection between gut microbiota, vitamin A deficiency, and ASD, suggesting that restoring the microbiota balance and addressing nutritional gaps could be crucial for managing ASD symptoms. In light of this evidence, it is important to consider specific corrective treatments for children with identified nutritional deficiencies or absorption issues, such as intravenous therapy when necessary. These findings support the notion that a personalized approach, based on proteomic analysis and nutritional intervention, could offer promising therapeutic potential for improving the health and behavior of children with ASD. Such interventions may enhance gut health, reduce neuroinflammation, and improve neurodevelopmental outcomes.

In turn, the study by Aagaard et al. [97] focused on the impact of high-dose vitamin D3 supplementation during pregnancy on the risk of neurodevelopmental disorders in children at the age of 10. The results of the study suggested potential benefits of vitamin D3 supplementation during pregnancy, which may reduce the risk of neurodevelopmental disorders in offspring. This could be of significant relevance when considering the role of vitamins in the treatment and prevention of brain development disorders. On the other hand, further research is needed to better understand the long-term effects and optimal dosages for such interventions.

## 4. Oxidative Stress in the Pathophysiology of ASD

Oxidative stress is a state of imbalance between the production of free radicals and the body’s ability to neutralize them through defense mechanisms [98,99]. Free radicals, especially ROS, are molecules containing unpaired electrons and are characterized by high chemical reactivity. Their excessive accumulation leads to cellular damage at the level of lipids, proteins, and genetic material, contributing to the development of numerous pathologies, such as cancer, cardiovascular diseases, and neurodegenerative disorders [99,100,101]. In the human body, free radicals are primarily produced as by-products of cellular respiration in mitochondria. During oxidative phosphorylation, a small fraction of electrons “leak” during electron transfer in the respiratory chain and react with oxygen, forming superoxide anion (O_2_•^−^) [100]. This radical can undergo further transformations, leading to the formation of hydrogen peroxide (H_2_O_2_), which in the presence of transition metal ions such as iron or copper converts into the highly toxic hydroxyl radical (OH•) via the Fenton reaction [102]. Similarly, reactive nitrogen species (RNS), such as nitric oxide (NO•), are generated through the action of enzymes like nitric oxide synthase (NOS) and can react with ROS to form peroxynitrite (ONOO^−^), which also causes cellular damage [103]. External factors, such as ultraviolet radiation (UV), environmental pollution, smoking, and toxins, further exacerbate ROS production by stimulating inflammatory processes and damaging cellular structures [100]. ROS, such as the OH•, initiate the oxidation of unsaturated fatty acids in cellular membranes, leading to the formation of unstable aldehydes like malondialdehyde (MDA). These products can act toxically, disrupting the function of biological membranes and the integrity of cellular organelles [99,101]. Free radicals can modify amino acid structures in proteins, leading to unnatural cross-links, denaturation, or fragmentation. An example is the oxidation of cysteine residues in enzymes, which can result in the loss of their catalytic activity [100]. ROS can cause deoxyribonucleic acid (DNA) strand breaks, oxidative modifications of nitrogenous bases, such as the formation of 8-oxoguanine, and abnormal mutations. Such damage increases the risk of carcinogenesis and other diseases associated with genetic mutations [99,102]. The human body possesses a multi-level defense system to protect against the effects of oxidative stress. These mechanisms are divided into enzymatic and non-enzymatic antioxidants. Among enzymatic antioxidants, superoxide dismutase (SOD) catalyzes the dismutation of O_2_•^−^ into H_2_O_2_ and oxygen (O_2_). Catalase (CAT) converts H_2_O_2_ into water (H_2_O) and oxygen, reducing its toxicity, while glutathione peroxidase (GPx) reduces H_2_O_2_ and organic hydroperoxides to H_2_O or corresponding alcohols using glutathione (GSH) as an electron donor [104]. Non-enzymatic antioxidants include vitamin C, which neutralizes ROS in aqueous environments, and vitamin E, which acts in lipid environments, protecting cellular membranes from peroxidation. GSH, a tripeptide with strong reducing properties, plays a key role in detoxifying ROS and regenerating damaged molecules [104]. Among natural compounds found in fruits and vegetables that chelate metal ions and neutralize free radicals are carotenoids and polyphenols [105]. Antioxidant mechanisms are transcriptionally regulated by the nuclear factor erythroid 2-related factor 2-Kelch-like ECH-associated protein 1 (Nrf2-Keap1) signaling pathway. In response to oxidative stress, the Nrf2 activates the expression of genes encoding antioxidant enzymes and detoxification proteins. This is a key element in cellular adaptation, allowing the more effective combating of oxidative stress [104]. When ROS production exceeds the capacity of defense mechanisms to neutralize them, damage accumulation and disease development occur. Chronic oxidative stress is particularly associated with aging, atherosclerosis, type 2 diabetes, Alzheimer’s disease, Parkinson’s disease, and numerous cancers [101].

Biomarkers of oxidative stress are indicators that help assess the level of oxidative stress in the body. These biomarkers are utilized in the diagnosis of numerous diseases associated with chronic inflammation, aging, diabetes, or cancer. They can also be used to monitor the effectiveness of antioxidant therapies or other medical interventions. Furthermore, the levels of these biomarkers can serve to evaluate the risk of chronic diseases or to predict their progression. Ultimately, the oxidative stress biomarkers provide critical information about the body’s health status and its ability to cope with free radicals and other reactive oxygen species. Key biomarkers include the products of lipid peroxidation, protein modifications, nucleic acid damage, as well as antioxidants and antioxidant enzymes. MDA and 4-hydroxy-2-nonenal (HNE) are commonly studied products of lipid peroxidation. MDA is generated during the oxidation of PUFA and is one of the most useful markers for monitoring oxidative damage. HNE, a product of omega-6 fatty acid peroxidation, acts as a “second messenger” of oxidative stress, influencing processes such as apoptosis and autophagy [106]. Lipid peroxidation products like MDA and HNE can form adducts with proteins, indicating oxidative damage. These adducts are used as biomarkers of oxidative stress-induced damage, especially in diseases such as atherosclerosis and diabetes [107]. Isoprostanes, derived from arachidonic acid, and neuroprostanes, formed from DHA, are widely employed as lipid peroxidation biomarkers and can be used to assess oxidative status and related diseases, such as neurodegenerative disorders and atherosclerosis [108]. Hydroxyoctadecadienoic acids, derived from linoleic acid during lipid peroxidation, can serve as indicators of oxidative stress in clinical studies and for evaluating the effectiveness of antioxidant therapies [109]. Advanced oxidation protein products (AOPPs) are derived from oxidized proteins, primarily albumin, but also fibrinogen and lipoproteins. This process results from oxidative stress, particularly in the presence of enzymes like myeloperoxidase that act in the presence of H_2_O_2_ and halides. AOPPs are mainly formed through the reaction of plasma proteins with oxidizing compounds such as hypochlorous acid. The formation of AOPPs involves carbonyl group modifications and cross-linking, such as dityrosine bridges that stabilize the resulting structures. Increased AOPP levels are observed in numerous pathological conditions, such as diabetes, chronic kidney diseases, and inflammation [110]. AOPPs are markers of oxidative stress that exacerbate inflammatory processes and may contribute to the progression of diseases like atherosclerosis, diabetic nephropathy, and chronic kidney diseases. Research suggests that AOPPs may directly activate monocytes and trigger inflammatory responses, especially in kidney diseases [111]. AOPPs are cleared from circulation primarily by the liver and spleen. Pharmacokinetic studies show that oxidized albumin, a major AOPP protein, rapidly exits circulation and is swiftly taken up by these organs [112]. Oxidative stress causes DNA damage, detectable through markers like 8-hydroxy-2′-deoxyguanosine (8-OHdG). This is one of the most frequently used tools for assessing oxidative damage in cells [113]. DNA damage induced by oxidative stress results from the action of ROS, which can lead to significant modifications in DNA structure. These changes have critical implications for cellular function and can result in mutations, cellular damage, and even cancer development. Oxidative stress induces DNA damage through the activation of enzymes like nucleases and the direct reaction of hydroxyl radicals with DNA, causing various changes, including base modifications, abasic sites (missing bases), and DNA strand breaks [114]. Mitochondrial DNA (mtDNA) is particularly susceptible to oxidative damage due to its proximity to respiratory chains, the main sources of ROS. This damage can lead to mtDNA degradation and loss of mitochondrial function, which may underlie the mechanisms of aging and diseases like macular degeneration [115]. DNA damage caused by oxidative stress is primarily repaired through base excision and nucleotide excision repair mechanisms. The cell’s ability to repair this damage is crucial for avoiding mutations and carcinogenesis [116]. Accumulated DNA damage from oxidative stress increases with age, contributing to cellular aging and a heightened risk of various diseases. mtDNA appears to be more vulnerable to such damage than nuclear DNA. It is well-established that oxidative stress can lead to DNA damage affecting cellular aging, the development of neurodegenerative diseases, and cancer. DNA repair mechanisms are vital for preventing mutations and maintaining genomic integrity [117,118].

Oxidative stress, associated with the overproduction of ROS and insufficient efficacy of antioxidant mechanisms, plays a key role in the pathophysiology of many diseases, including ASD. Numerous scientific studies have indicated that individuals with ASD are more susceptible to oxidative stress, which can lead to cellular and tissue damage, particularly in the central nervous system. A meta-analysis of eighty-seven studies showed that children with ASD exhibit significantly higher levels of oxidative stress markers compared to neurotypical children. For example, elevated levels of MDA, a product of lipid peroxidation indicating cell membrane damage; homocysteine, a compound whose high levels can damage blood vessels and exacerbate oxidative stress; nitric oxide, whose excessive production may lead to the formation of RNS, further intensifying oxidative stress; and copper, whose increased levels are linked to the overproduction of ROS, were all observed. At the same time, reduced levels of GSH, a key antioxidant, highlight the weakening of the body’s natural defense mechanisms. GSH plays a leading role in neutralizing ROS and detoxifying the body, and its deficit is a clear indicator of antioxidant dysfunction. Lower levels of vitamins such as B9, B12, D, and E have also been noted [119]. Antioxidant system dysfunction in individuals with ASD has been demonstrated through elevated levels of antioxidant enzymes, such as superoxide dismutase (SOD1). Although SOD1 is responsible for breaking down superoxide anions, its excess may paradoxically serve as a marker of chronic oxidative stress, for which the body attempts to compensate. Studies showing a correlation between elevated SOD1 levels and the severity of ASD symptoms have suggested that disrupted oxidative balance may influence ASD neurobiology and progression. Furthermore, other enzymes, such as CAT and GPx, also exhibit altered activity in individuals with ASD [120]. The dysfunction of the antioxidant system in individuals with ASD has been confirmed by elevated SOD levels. SOD1 is primarily located in astrocytes, particularly in the cytosol, while superoxide dismutase 2 (SOD2) is predominantly found in neuronal mitochondria [121]. Excess SOD may paradoxically mark chronic oxidative stress, for which the body attempts to compensate. Additionally, differences in SOD1 and SOD2 levels have been observed depending on sex, with males exhibiting higher levels of both markers [120]. 3-nitrotyrosine (3-NT) is an indicator of reactive nitrogen species. Its presence indicates redox imbalance in the body. Elevated levels of 3-NT are associated with various diseases, including neurodegenerative disorders, cardiovascular diseases, inflammatory conditions, and cancers. In a study by Sajdel-Sulkowska et al. [122], a significant increase in 3-NT was observed in specific brain regions of individuals with ASD, including the orbitofrontal cortex, Wernicke’s area, cerebellum, caudate nucleus, and pons. Elevated 3-NT levels were linked to areas responsible for speech, emotions, social behavior, and sensory–motor coordination. Moreover, high levels of 3-NT may disrupt the structure and physiological function of many proteins, potentially leading to their loss of function [123,124]. A critical factor is neurotrophin-3 (NT-3), synthesized and secreted by brain nerve cells and cells dependent on peripheral sensory, motor, and sympathetic neurons. This protein supports survival, differentiation, maturation, synapse formation, and neuron growth [125]. Sajdel-Sulkowska et al. observed a correlation between 3-NT and NT-3 levels in the cerebellum of individuals with autism. This effect manifested as increased oxidative stress and NT-3 overexpression, leading to cerebellar hypertrophy. Interestingly, in other brain regions, the correlation was reversed, with increased oxidative stress causing decreased NT-3 expression, resulting in cell death and exacerbated neurodegenerative changes [122]. Another study revealed reduced GPx and GSH levels, elevated lipid peroxidation products, and both reduced and elevated SOD levels in individuals with ASD. These individuals may therefore have diminished detoxification capabilities and heightened vulnerability to oxidative stress [126]. Additionally, increased susceptibility to infections, difficulty mitigating inflammation, and reduced capacity for environmental pollutant detoxification were observed in individuals with ASD [126].

In studies conducted on children with autism (36 boys and 9 girls) aged 3–11 years, decreased levels of GSH were observed along with elevated levels of MDA, 8-OHdG, and CAT. These changes are associated with the body’s reduced ability to neutralize ROS and an increased rate of cell breakdown, including neuronal cells. Neuronal loss has been observed in the cerebellum [127], and stereological studies have also indicated significant losses in the amygdala area [128]. Mitochondria are the primary source of ROS and are responsible for their neutralization. Mitochondrial dysfunction in ASD leads to excessive ROS production, resulting in oxidative stress. The research has indicated that children with autism exhibit increased mitochondrial activity, making them more susceptible to oxidative stress. Mitochondria are also crucial for energy production in cells. In children with ASD, disturbances in energy generation have been observed, which may affect brain function, including neurotransmission processes and synaptic plasticity. Mitochondrial dysfunction may further be exacerbated by the influence of gut microbiota. Changes in the gut microbiota have been observed in ASD, which can affect mitochondria through the production of toxic metabolites such as propionic acid and butyric acid. These metabolites act as mitochondrial fuel. Mitochondrial dysfunction in ASD may alter the way these metabolites are processed, leading to further oxidative stress and cellular dysfunction. Mitochondria play an important role in cell repair; thus, their dysfunction can lead to increased susceptibility of brain cells to oxidative damage, which in turn may influence the development of ASD [129]. The authors of the studies also emphasized the impact of environmental factors on the occurrence and development of ASD. Exposure to environmental toxins such as heavy metals (e.g., mercury, lead) and chemicals may increase ROS production, which in turn leads to oxidative stress in the brain. Increased vulnerability to oxidative stress in individuals with ASD may stem from an inability to effectively eliminate these toxins [130]. Individuals with ASD often exhibit elevated levels of oxidative stress and inflammation, which may contribute to the exacerbation of symptoms. Therapeutic interventions for ASD encompass various approaches to alleviate symptoms and improving quality of life. These include antioxidant support, appropriate diet, anti-inflammatory therapies, nutraceuticals, and modifications in gut microbiota. The use of vitamin C, E, GSH, and nutraceuticals influences the regulation of antioxidant mechanisms [131]. A decrease in vitamin C levels promotes the development and progression of many diseases, including neurological disorders. This deficiency results in reduced growth and weakened neuronal development, increasing their susceptibility to oxidative stress-induced damage [132].

Vitamin E integrates into cell membranes, acting as an effective inhibitor of lipid peroxidation by neutralizing free radicals. This reaction forms oxidized derivatives of vitamin E, which regain their activity through reduction back to their original form. This process relies on interactions between water- and fat-soluble substances via enzymatic and non-enzymatic mechanisms [133]. A study of fifty-one children with ASD noted deficiencies in vitamins critical to the body’s antioxidant defense mechanisms, particularly vitamins E and C, which protect cells from damage caused by free radicals and oxidative stress. The authors highlighted that these deficiencies are primarily attributed to the inadequate diets of children with ASD, which are often low in these vitamins. The dietary specificity in ASD is frequently shaped by sensory difficulties, dietary restrictions, or preferences, complicating the provision of adequate nutrients. These findings underscore the need for careful dietary planning and supplementation in this population to enhance health and functioning [133].

Research on GSH supplementation in children with ASD revealed lower levels of reduced plasma GSH and higher levels of oxidized glutathione (GSSG) compared to typically developing children [134,135]. Kern et al. reported a reduction of up to 20–40% [134]. Studies have suggested potential benefits from using antioxidant supplements, such as GSH and *N*-acetylcysteine (NAC), in treating children with ASD. GSH supplementation, whether oral or transdermal, increased metabolites associated with trans-sulphuration (e.g., cysteine, taurine, sulfates) but did not significantly affect total blood GSH levels. NAC, a GSH precursor, demonstrated promising effects in reducing irritability and self-injurious behaviors in children with severe behavioral symptoms. Case studies noted significant reductions in irritability scale scores, with good tolerability and minimal side effects, primarily GI. NAC’s mechanism likely involves mitigating oxidative stress, making it a promising therapeutic support [135].

Mouse model studies of ASD induced by prenatal valproic acid (VPA) administration have suggested the potential benefits of astaxanthin as a therapeutic agent. Astaxanthin significantly increased the frequency of social interactions (e.g., sniffing other mice) and time spent in social contact during behavioral tests. Open-field tests showed reduced anxiety and improved overall activity in mice treated with astaxanthin. This substance enhanced response to pain stimuli, suggesting its positive impact on sensory perception. Additionally, astaxanthin lowered markers of oxidative stress, such as MDA, NO•, and APOP, while enhancing enzymatic antioxidants (CAT and SOD) and GSH levels in the brain and liver [136].

Other substances with promising properties for improving cognitive functions and reducing inflammation include resveratrol, omega-3 fatty acids, and green tea extracts [131]. The DHA is essential for cognitive functions, neuronal growth, neurotransmission, and membrane fluidity. Its deficiency in ASD may lead to the overproduction of pro-inflammatory cytokines, oxidative stress, and neurotransmitter metabolism disturbances [137]. DHA supplementation has shown potential in reducing symptoms such as stereotypical behaviors, hyperactivity, and social difficulties. Studies have suggested improvements in concentration, motor skills, and language development in children with ASD during supplementation. The gut microbiota influences brain function through the production of SCFAs and the regulation of immune responses. In ASD, increased gut permeability and microbial imbalance can exacerbate neuroinflammation. DHA may support gut barrier functions by reducing permeability and strengthening epithelial resistance to inflammatory factors. Additionally, DHA promotes the growth of beneficial bacteria, such as Bifidobacterium and Lactobacillus, which support intestinal epithelial integrity and reduce inflammation. Animal model studies have suggested that combining DHA with probiotics may improve gut health, reducing the behavioral symptoms in ASD [137]. Table 1 summarizes examples of substances with studied positive therapeutic properties.

## 5. The Role of Trace Elements in ASD

Trace elements play a crucial role in neuropsychiatric health, and their balance in the body is essential for the proper functioning of the nervous system. Deficiencies in zinc, iron, copper, and magnesium have been linked to numerous neuropsychiatric disorders. The research has indicated that children with tic disorders have lower levels of zinc, iron, and copper compared to control groups, suggesting that these elements may be involved in the pathogenesis of tic disorders [144]. In children with ASD, a relationship has been observed between trace element levels, such as manganese and copper, and neuroinflammatory markers. Elevated levels of manganese and copper may exacerbate inflammatory responses in the brain, potentially influencing the severity of ASD symptoms [145]. Zinc deficiency and copper excess have been shown to disrupt neurotransmitter functions, which are associated with depression and anxiety disorders. These trace elements play a vital role in regulating the neurochemical processes in the brain [146]. In older adults, imbalances in trace elements such as calcium, copper, and cadmium impact cognitive functions. High levels of cadmium and copper correlate with cognitive decline, whereas higher calcium levels have protective effects [147]. Exposure to toxic elements like lead, mercury, and arsenic causes severe neurotoxic damage, particularly during early development. These substances can alter the brain’s structure and function, leading to long-term effects on neuropsychiatric health [148].

### 5.1. Zinc

Zinc is crucial for many physiological processes, including the functioning of the immune system, GI health, and neurological development. It exhibits antioxidant and anti-inflammatory properties but is not stored in the body, requiring regular dietary intake. Zinc is a component of many metalloenzymes from the oxidoreductase, hydrolase, and ligase groups [149]. SOD constitutes a fundamental element of the body’s antioxidant defense system and is key to maintaining a balance between the production and neutralization of free radicals. Zinc is an essential component of the active form of this enzyme. This element is present in cytoplasmic SOD1 (CuZn-SOD) and extracellular superoxide dismutase 3 (EC-SOD) [150]. Zinc is a vital micronutrient that plays key roles in the body, including in the immune, nervous, and metabolic systems. Zinc deficiency is particularly common in individuals with ASD due to selective eating and other health issues, potentially exacerbating autism symptoms. It is a micronutrient that supports immune system functions, GI development, and neurological health. Zinc deficiency can lead to worsened symptoms such as oxidative stress, inflammation, and gut dysbiosis, often present in individuals with ASD. Zinc deficiency may also affect metabolic disorders, including type 2 diabetes, insulin resistance, and obesity, which frequently co-occur with ASD [151,152]. Zinc is essential for taste and smell perception. Individuals with ASD often experience selective eating, leading to a poor diet and resulting in deficiencies in zinc and other micronutrients. Factors such as a zinc-deficient diet, GI disorders, and lifestyle choices can increase the risk of zinc deficiency. The processes of zinc absorption and elimination also depend on the quality and type of food consumed [151]. Zinc plays a crucial role in synaptic function, particularly in regulating SHANK proteins (scaffolding proteins that regulate the formation, organization, and plasticity of excitatory synapses), which are essential for organizing excitatory synapses. These proteins are involved in autism development, and their function depends on zinc binding. Zinc deficiency leads to dysfunction in these proteins, affecting proper neural signal transmission [153]. Zinc is also involved in modulating NMDA receptors, which are key for memory processes and synaptic plasticity. Imbalances in zinc levels can lead to the dysfunction of these receptors, relevant to neuropsychiatric disorders such as autism [154]. Zinc is essential for neutralizing oxidative stress in the brain, which is critical for preventing neuronal damage. Its deficiency may increase vulnerability to damage caused by free radicals [151]. Zinc deficiency has been linked to autism development. Studies have shown that children with ASD often have lower zinc levels compared to control groups. This deficiency may negatively impact brain development and synaptic functions, contributing to ASD symptoms [155]. Zinc deficiency also disrupts the functioning of SHANK proteins, which are essential for proper synapse functioning. Mutations in SHANK genes (particularly SHANK3) are associated with ASD. It has been established that zinc supplementation can improve synaptic functions in animal models of autism [156]. In studies by Fourie et al. [157], the effects of zinc supplementation on mitigating ASD-like symptoms and synaptic dysfunction in mice with mutations in the SHANK3 protein gene were analyzed. Zinc supplementation reduced repetitive behaviors, anxiety symptoms, and difficulties in recognizing social novelty in mice with SHANK3 mutations, bringing their behavior closer to control groups. Higher zinc levels in the diet improved synaptic transmission and plasticity in cortico-striatal synapses, particularly through the modulation of NMDA receptor function and recruitment of zinc-sensitive SHANK2 protein. Zinc supplementation is believed to contribute to improved synaptic function and a reduction in autism symptoms [157].

In summary, zinc plays a crucial role in brain function and may significantly impact autism development, especially in cases of deficiency. The mechanisms of zinc action include the regulation of synaptic proteins, neurotransmission, and protection against oxidative stress.

### 5.2. Copper

Copper is a key trace element in the body, playing a vital role in numerous biological processes. One of its most important aspects is its involvement in catalytic activity. Copper acts as a cofactor for enzymes participating in redox reactions, such as cytochrome oxidase and superoxide dismutase, supporting cellular respiration and protection against oxidative stress [158]. This element is essential for neurotransmitter synthesis and the development of the central nervous system. Disruptions in its metabolism are associated with numerous diseases, including neurodegenerative disorders such as Wilson’s disease and Alzheimer’s disease [159]. As a component of enzymes like superoxide dismutase, copper helps neutralize free radicals, preventing damage to DNA and cell membranes [152]. Moreover, it is crucial for supporting the absorption and transport of iron in the body, which is essential for proper hemoglobin production [160]. The application of copper in biomaterials has led to significant antibacterial, angiogenic, and osteogenic properties, which support wound healing and bone regeneration [161]. Excess copper can lead to toxicity, partly due to the generation of reactive oxygen species. The processes of transport, storage, and excretion of copper are precisely regulated by transport and chaperone proteins, such as copper-transporting P-type ATPases [162]. Copper, as a trace element, also supports the complexation process via the presence of metallothioneins, which bind heavy metals like cadmium and lead, reducing their toxicity [163]. In the presence of heavy metals, copper helps activate pathways related to tolerance and detoxification, including the synthesis of phytochelatin peptides that bind and neutralize toxic metals [164]. Considering the broad significance of copper in the functioning of the body and the maintenance of its homeostasis, it becomes particularly important to understand its potential impact on the etiology of various diseases, including ASD. The importance of this element extends beyond basic biological processes, as its deficiency or excess may play a role in the pathological mechanisms of numerous conditions. In this context, analyzing the involvement of copper in the development and progression of ASD emerges as a crucial area of research, shedding light on the potential connections between micronutrient balance and mental and neurological health. The research has indicated that children with autism often have higher plasma copper levels and lower zinc levels. The zinc-to-copper ratio (Zn/Cu) is frequently reduced, suggesting disruptions in metal homeostasis and metallothionein function, which may contribute to ASD development [165]. High copper levels can impact the excitatory synaptic function by disrupting the homeostasis of metal ions like zinc. These disruptions may influence the pathogenesis of ASD through dysfunctions in SHANK proteins, which are crucial for proper synaptic functioning in the brain [166]. Furthermore, copper overload may lead to neurotoxicity, particularly in children with ASD. High copper levels combined with zinc deficiency can increase oxidative stress in the brain and negatively affect nervous system development [167]. Curtin et al. [168] suggested that the risk of ASD might be predicted by observing disruptions in the metabolic cycle of zinc and copper during the prenatal period. The authors also noted that it is not the concentrations but the rhythmicity of metals in this dynamic system that is linked to ASD risk. It is evident that both high concentrations of heavy and trace metals could be responsible for the etiology of ASD [168].

In summary, copper plays a crucial role in the body’s metabolic processes, and its excess may be linked to neuropsychiatric disorders such as autism. Both disruptions in copper homeostasis and its interactions with zinc are significant factors influencing the development of ASD.

### 5.3. Selenium

Selenium is an essential nutrient that plays a crucial role in maintaining health at both cellular and systemic levels. An adequate dietary intake of selenium is necessary to support various physiological processes, including protection against oxidative stress, regulation of thyroid hormones, enhancement of immunity, and prevention of chronic diseases. However, both selenium deficiency and excess can be harmful, emphasizing the importance of maintaining a balance in its intake. Selenium is a key micronutrient essential for the proper functioning of the body, influencing numerous metabolic, protective, and regulatory processes. It is a component of selenoproteins such as GPx, thioredoxin reductase, and selenoprotein P, which protect cells from oxidative stress by neutralizing ROS and free radicals [169]. Selenium plays a significant role in the development and activity of T lymphocytes and antibody production, strengthening the immune system and enhancing resistance to infections [170]. It also participates in thyroid hormone metabolism, enabling the conversion of thyroxine (T4) to active triiodothyronine (T3), which impacts metabolism regulation and overall hormonal balance [171]. Due to its antioxidant and anti-inflammatory properties, selenium may reduce the risk of cardiovascular diseases such as atherosclerosis and hypertension [172]. In terms of reproductive health, selenium supports the functioning of the reproductive system, particularly in men, by improving sperm motility and fertilization capacity [173]. Studies have suggested that selenium may decrease the risk of certain cancers, including liver, prostate, and colorectal cancers, through apoptosis regulation and antioxidant activity [174]. Beyond its antioxidant role, selenium is involved in neurotransmission and neuroprotection, processes in which selenoproteins are integral components. These proteins are essential for proper neuronal function, particularly in dopaminergic and GABAergic neurotransmission. Selenium may influence cognitive and motor functions as well as coordination through its effects on these pathways [175]. It plays an essential role in regulating neuroprotective mechanisms, particularly in response to oxidative damage and inflammation. Selenoproteins, such as selenoprotein P, act as “survival factors” for neurons, protecting them from damage and cell death [176]. Many selenoproteins are expressed in various brain regions. The known and described selenoproteins include not only selenoprotein P but also F, H, I, K, M, N, O, R, S, T, V, and W [177].

In a study by Wu et al. [178], plasma selenium concentrations were compared in a group of 92 autistic children and a control group of 103 individuals. Significant statistical differences were found between the groups, with selenium concentrations in plasma being lower in autistic children than in the control group. Zinc concentrations, also measured in this study, were similarly lower in the autistic group. It is suggested that oxidative stress, which reduces the functional capacity of selenoproteins, may form the basis of the association between ASD and selenium levels. Impaired antioxidant function of selenoproteins leads to altered gene expression, resulting in the formation of abnormal neural connections [177]. It is also important to consider the variability in selenium levels, with both excess and deficiencies observed. These differences are believed to stem from the use of different methods to measure selenium levels and the evaluation of various clinical materials such as blood, urine, or hair [145]. An intriguing observation is that maternal selenium deficiency during pregnancy may increase the risk of neuropsychiatric disorders in children and contribute to the development of ASD. This may be due to the effect of low selenium levels during the prenatal period on fetal brain development [179].

Selenium plays a key role in nervous system function, and its deficiencies can lead to increased oxidative stress, contributing to the development of ASD and other neuropsychiatric disorders.

### 5.4. Other Trace Elements

Among other elements associated with ASD, iron plays a crucial role. It is key to brain development, supporting cognitive, motor, and behavioral functions. Its deficiency, especially during the prenatal period, may lead to neurological disorders associated with an increased risk of ASD. The research has suggested that disruptions in iron metabolism may be a contributing factor to the pathogenesis of ASD [180]. Numerous studies have shown that children with ASD often have iron deficiency and low ferritin levels, which may result in anemia. The findings have suggested that low ferritin levels are common in children with ASD and may influence symptoms such as cognitive and behavioral problems [181,182,183]. Children with ASD frequently have specific dietary preferences and selective eating habits, which may lead to insufficient iron intake. Dietary restrictions related to ASD, such as avoiding iron-rich foods, are associated with more frequent occurrences of iron deficiencies and anemia, potentially exacerbating existing behavioral issues [184]. Iron supplementation in children with ASD may be an effective intervention for reducing anemia symptoms and improving cognitive and behavioral functions. The research has suggested that ferritin level assessment and iron supplementation should be standard care for children with ASD [185].

Magnesium is another crucial element in the body. Studies by Strambi et al. [186] found that children with ASD have lower plasma magnesium concentrations compared to healthy children, highlighting the necessity of monitoring and potentially enriching their diets, especially when these children exhibit tendencies to select certain foods while excluding others. Such behaviors may lead to deficiencies in specific amino acids and proteins. Since most magnesium in the blood is protein-bound, monitoring and supplementation can play a vital role in supporting appropriate diet planning for children with ASD [186,187]. Research by Józefczuk et al. [188] revealed very low magnesium levels in the hair of children with ASD, despite serum magnesium levels being within the normal range. The reduced magnesium concentration in hair may result from environmental pollution, cosmetic use that depletes magnesium, and diseases (including neurological ones) that affect magnesium intake and absorption [188,189,190]. Evidence shows that supplementing magnesium is particularly effective when combined with vitamin B6. A study involving 50 children with ASD aged 2 to 12 demonstrated improvements in cognitive and emotional functions in the group receiving magnesium and vitamin B6 supplementation compared to the placebo group [191]. In a study of 1020 children with ASD, low magnesium levels were negatively correlated with the severity of core ASD symptoms, such as communication issues and motor impairments [192]. Moreover, differences in magnesium levels across studies may stem from variations in methodologies and research populations. Nonetheless, it is inferred that low magnesium levels, alongside other element deficiencies, impact synaptic functioning, which may be significant in the etiology of ASD [193].

The levels of trace elements have a significant impact on the functioning of antioxidant enzymes, which depend on them. Dysfunctions in this area may contribute to the development and manifestation of ASD. Maintaining a proper balance of trace elements is crucial for the effective operation of the body’s defense mechanisms against oxidative stress, which plays a significant role in the etiology of ASD.

Because the pathophysiology of ASD is influenced by oxidative stress and inflammation, attention is drawn to the intermediary role of the AhR and PPARγ. The AhR receptor is a key regulator of oxidative balance in cells by modulating reactive oxygen species (ROS) levels and the expression of antioxidant enzymes such as SOD, CAT, and GPx. AhR controls ROS levels through the induction of cytochrome P450 enzymes (CYP1A1). Upon ligand activation, AhR induces the expression of genes encoding cytochrome P450 enzymes, such as CYP1A1, CYP1A2, and CYP1B1. These enzymes participate in xenobiotic metabolism, which may lead to the generation of ROS as by-products [194,195,196]. AhR regulates the expression of key antioxidant enzymes through transcriptional mechanisms and signaling pathways. The primary regulator of the antioxidant response is the Nrf2. Activation of AhR may facilitate the release of Nrf2 from the Keap1 complex, leading to its translocation into the nucleus and the induction of genes encoding antioxidant enzymes such as SOD, CAT, and GPx. Joint activation of AhR and Nrf2 enhances the antioxidant response in cells exposed to various substances, including xenobiotics [197,198]. Exposure to AhR ligands, such as dioxins, polycyclic aromatic hydrocarbons (PAHs), and other environmental pollutants, is associated with increased ROS levels. These substances can activate AhR, causing excessive ROS production and oxidative damage [199]. Environmental compounds activating AhR are often mentioned as potential risk factors in ASD, suggesting that AhR dysregulation may play a role in the pathophysiology of this disorder [200]. Natural AhR ligands, such as flavonoids or tryptophan metabolites, have the potential to modulate AhR in a manner that is beneficial to oxidative and immune balance. Drugs or supplements that enhance Nrf2 activity could synergize with AhR to reduce oxidative stress and inflammation in ASD [201,202].

The second important receptor is PPARγ, a ligand-dependent transcription factor belonging to the nuclear receptor family. PPARγ is primarily known for its role in lipid metabolism and energy homeostasis regulation, but it also plays a key role in modulating oxidative stress, inflammation, and nervous system function. Its functioning is particularly interesting in the context of neurodevelopmental disorders such as autism (ASD). This receptor reduces oxidative stress by inducing antioxidant enzymes such as SOD, CAT, and GPx [203]. PPARγ inhibits the activity of ROS-generating enzymes such as nicotinamide adenine dinucleotide phosphate (NADPH) oxidase and cytochrome P450 enzymes, which may lead to excessive ROS production [204]. Elevated ROS levels and decreased antioxidant enzyme activity are observed in individuals with ASD, leading to oxidative damage in neurons. PPARγ activation can mitigate these effects by enhancing the antioxidant response [204].

**Figure 2:**
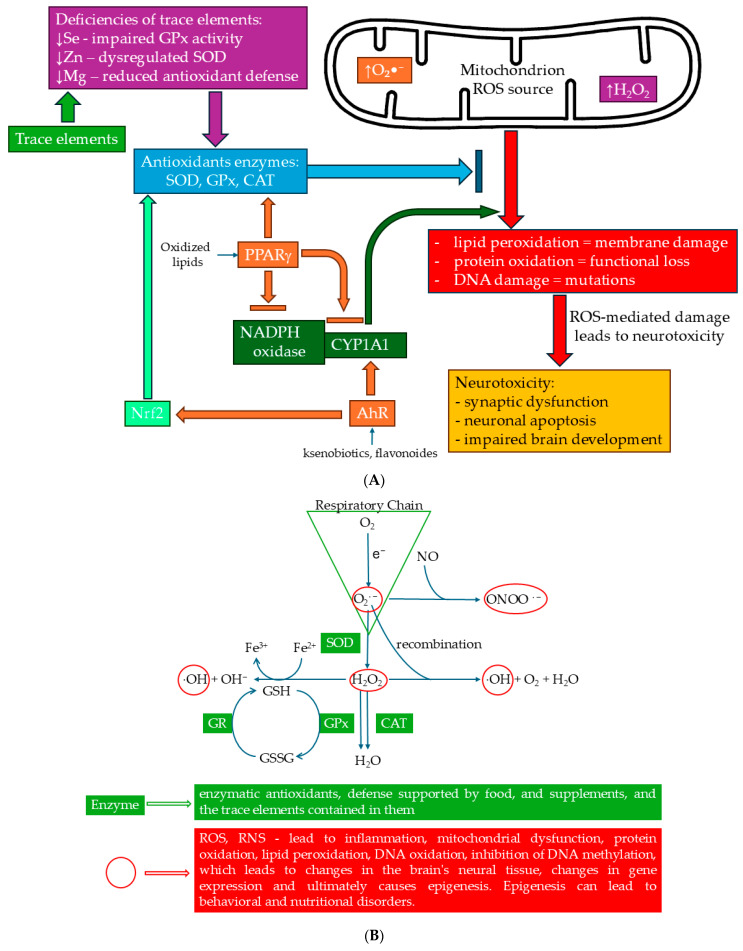
(**A**). Relationships between trace elements, antioxidants, and ROS in relation to ASD. Abbreviations: aryl hydrocarbon receptor (AhR); catalase (CAT); cytochrome P450 (CYP1A1); deoxyribonucleic acid (DNA); glutathione peroxidase (GPx); hydrogen peroxide (H_2_O_2_); magnesium (Mg); nicotinamide adenine dinucleotide phosphate (NADPH); nuclear factor erythroid 2-related factor 2 (Nrf2); peroxisome proliferator-activated receptor gamma (PPARγ); reactive oxygen species (ROS); selenium (Se); superoxide anion (O_2_•^−^); superoxide dismutase (SOD); zinc (Zn). The arrow ending with a perpendicular beam indicates inhibition. (**B**). Oxidative stress mechanisms relevant to ASD. Abbreviations: catalase (CAT); deoxyribonucleic acid (DNA); reduced glutathione (GSH), oxidized glutathione (GSSG), glutathione peroxidase (GPx); glutathione reductase (GR); hydrogen peroxide (H_2_O_2_); reactive oxygen species (ROS); reactive nitrogen species (RNS); superoxide anion (O_2_•^−^); superoxide dismutase (SOD).

Gut microbiota influences PPARγ activation through the production of short-chain fatty acids (SCFAs), such as butyrate, which are natural agonists of PPARγ. Gut dysbiosis, often observed in individuals with ASD, may reduce PPARγ activation and exacerbate oxidative stress and inflammation [205]. Synthetic PPARγ agonists, which exhibit anti-inflammatory and antioxidant effects, may improve mitochondrial function and reduce oxidative stress, showing promise for improving ASD outcomes [206,207]. Figure 2A shows the general pathways of connections between trace elements, antioxidant enzymes, ROS and AHR and PPARγ receptors in the aspect of ASD pathophysiology. Figure 2B presents the mechanisms of oxidative stress in the context of ASD.

## 6. Summary of Current Research

Recent studies have indicated that diet plays a significant role in the management of various health conditions, including ASD. One of the most discussed dietary interventions is the GFCF diet and KD. The evidence suggests that this diet may help alleviate some symptoms of ASD, although its effectiveness varies depending on the individual characteristics of patients. Supplementation with antioxidants and a diet enriched with trace elements can also help limit disease progression, its development, and alleviate symptoms. Trace elements and antioxidants support this by reducing oxidative stress. Some results of recent research were presented in Table 2.

## 7. Conclusions

Oxidative stress plays a key role in the pathology of ASD, as evidenced by elevated markers such as malondialdehyde and reduced levels of antioxidants like glutathione in affected individuals. This imbalance highlights the therapeutic potential of antioxidant interventions, including supplementation with glutathione and vitamins C and E, in mitigating symptoms. Trace elements such as zinc, selenium, and copper are equally crucial for neurodevelopment and oxidative stress regulation. Zinc deficiency and copper excess are particularly linked to synaptic dysfunction and heightened oxidative stress, exacerbating ASD symptoms, while selenium’s role in antioxidant defense underscores its importance in managing oxidative stress-driven neuroinflammation.

Specialized dietary interventions incorporating trace elements, omega-3 fatty acids, and probiotics offer promising strategies for addressing both behavioral and physiological symptoms of ASD. Modulating the gut–brain axis through probiotics, prebiotics, and fecal microbiota transplantation has demonstrated potential in alleviating gastrointestinal issues, stereotypical behaviors, irritability, and neuroinflammation while improving sleep quality, socialization, and communication skills. Omega-3 fatty acids, in particular, have shown promise in reducing neuroinflammatory responses and improving behavioral outcomes, reinforcing the significance of dietary strategies in ASD management. However, the significant heterogeneity of autistic behaviors, dietary habits, and nutritional statuses among patients necessitates well-controlled, large-scale clinical trials. Such studies should include comprehensive morphological and microbiome analyses to better understand the interplay between oxidative stress, dietary interventions, and behavioral outcomes. Detailed statistical analyses of these data could enable the development of individualized patient profiles, facilitating more tailored and effective therapeutic approaches. By addressing oxidative stress, neuroinflammation, and gut dysbiosis collectively, these interventions could significantly enhance the quality of life for both patients and their families.

## Figures and Tables

**Table 1:** Therapeutic substances studied for ASD.

Substance	Mechanism of Action	Therapeutic Effects	Ref.
Coenzyme Q10 (CoQ10)	Reduces oxidative stress	Improves oxidative balance, sleep quality, and GI issues	[138]
Camel Milk	Natural antioxidant supporting antioxidant enzymes	Reduces oxidative stress, improves behavioral symptoms	[139]
Melatonin	Regulates sleep and has antioxidant properties	Improves sleep quality and overall health	[140]
*L*-carnitine, luteolin, quercetin	Antioxidant and anti-inflammatory	Improves behavioral symptoms	[141]
High-antioxidant cocoa	Neutralizes free radicals	Reduces irritability, stereotypical behaviors, and improves communication	[142]
Vitamin C and NAC	Neutralizes free radicals, supports the immune system	Improves antioxidant status	[143]

Abbreviations: autism spectrum disorders (ASD); coenzyme Q10 (CoQ10); gastrointestinal (GI); *N*-acetylcysteine (NAC).

**Table 2:** A summary of current research on the relationship between ASD and diet and/or supplementation.

Diet/Supplementation	Therapeutic Effects	Ref.
The GFCF diet	Minimal changes in behavioral disorders after the GFCF diet	[21]
Modified KD/GF/MCT	50% of subjects demonstrated significant improvements in ASD behavior defined by 4 points or greater improvement in ADOS-2 total score. Also, potential biochemical effects of the diet, such as increased HDL, increased eosinophil percent, and lower white blood cell count were identified.	[35]
The KD diet	The diet proved to be effective in diminishing various generalized behaviors as it allowed the patients to concentrate better and increase their learning abilities.	[36]
The KD diet	The study showed that the improvements in ASD behavior after KD treatment were accompanied by alterations in mitochondria-related metabolites and trace elements, highlighting the role of metabolism in this neurological disorder.	[37]
The prebiotic galacto-oligosaccharide mixture (B-GOS^®^)	Administration of 1.8 g powder (80% of GOS content) during 6-week period. The improvement in anti-social behavior, significant changes in gut microbiota composition, and significant changes in fecal and urine metabolites.	[52]
Microbiota Transfer Therapy	At the end of treatment, an approximately 80% reduction in GI symptoms including significant improvements in symptoms of constipation, diarrhea, indigestion, and abdominal pain, was revealed. Also, behavioral ASD symptoms improved significantly and remained improved 8 weeks after treatment ended.	[53]
Probiotic supplement composed with *Lactobacillus plantarum*	Administration of 4.5 × 10^10^ CFU per capsule per day for 3 weeks during the 12 weeks study duration improved behavioral scores and the stool consistency, increased Enterococci and Lactobacilli group, decreased *Clostridium* cluster XIVa.	[54]
Omega-3 fatty acids supplementation	Administration of 1.5 g/d of omega-3 fatty acids demonstrated beneficial effects on behavior, reducing hyperactivity and stereotypical behaviors.	[70]
Zinc supplementation using mouse models	There is a tight relationship between ASD-like behavior and prenatal zinc deficiency in mice. Meta-analysis concluded that control of local zinc concentrations at synapses through zinc transporters may be a promising approach, and synaptic zinc transporters may be an interesting drug target.	[156]
Vitamin C and NAC	Improved antioxidant status, reduced irritability, and self-injurious behaviors.	[135]
Melatonin	Improved sleep quality and overall health.	[140]
Astaxanthin	Reduced oxidative stress markers, improved social behavior, and reduced anxiety in mouse models of ASD.	[136]
High-Antioxidant Cocoa	Reduced irritability and stereotypical behaviors, improved communication.	[142]
Magnesium and Vitamin B6 Supplementation	Improved cognitive and emotional functions in children with ASD.	[191]
Selenium Supplementation	Enhanced antioxidant functions, reduced oxidative stress, and prevented inflammatory conditions in ASD.	[178]
Iron Supplementation	Reduced anemia, improved cognitive and behavioral functions in children with ASD.	[185]

Abbreviations: Autism Diagnostic Observation Schedule, 2nd Edition (ADOS-2); autism spectrum disorder (ASD); bifidobacterium-enriched galacto-oligosaccharides (B-GOS); colony-forming unit (CFU); gluten-free (GF); gluten-free and casein-free (GFCF) diet; gastrointestinal (GI); high-density lipoprotein (HDL); ketogenic diet (KD); medium-chain triglycerides (MCT); *N*-acetylcysteine (NAC).

## Abbreviations

3-NT	3-nitrotyrosine
HNE	4-hydroxy-2-nonenal
8-OHdG	8-hydroxy-2′-deoxyguanosine
ATP	adenosine triphosphate
AOPP	advanced oxidation protein products
ADHD	attention deficit hyperactivity disorder
ADOS-2	Autism Diagnostic Observation Schedule, 2nd edition
ASD	autism spectrum disorder
CAT	catalase
CARS	Childhood Autism Rating Scale
CoQ10	coenzyme Q10
DNA	deoxyribonucleic acid
DSM-5	Diagnostic and Statistical Manual of Mental Disorders, fifth edition
DHA	docosahexaenoic acid
EPA	eicosapentaenoic acid
EC-SOD	extracellular superoxide dismutace
FMT	fecal microbiota transplantation
GABA	gamma-aminobutyric acid
GI	gastrointestinal
GSH	glutathione
GPx	glutathione peroxidase
GFCF	gluten-free and casein-free
AhR	aryl hydrocarbon receptor
H_2_O_2_	hydrogen peroxide
OH•	hydroxyl radical
IL-10	interleukin-10
IL-6	interleukin-6
KEAP1	Kelch-like ECH-Associated Protein 1
KD	ketogenic diet
LPS	lipopolysaccharides
MDA	malondialdehyde
MCT	medium-chain triglycerides
mtDNA	mitochondrial DNA
NAC	*N*-acetylcysteine
NT-3	neurotrophin-3
NADPH	nicotinamide adenine dinucleotide phosphate
NO•	nitric oxide
NOS	nitric oxide synthase
NMDA	*N*-methyl-*D*-aspartate
Nrf2	nuclear factor erythroid 2-related factor 2
GSSG	oxidized glutathione
PPARγ	peroxisome proliferator-activated receptor gamma
ONOO^−^	peroxynitrite
PUFA	polyunsaturated fatty acid
RCT	randomized controlled trial
RNS	reactive nitrogen species
ROS	reactive oxygen species
SCFA	short-chain fatty acid
O_2_•^−^	superoxide anion
SOD	superoxide dismutase
SOD1	superoxide dismutase 1
SOD2	superoxide dismutase 2
T4	thyroxine
T3	triiodothyronine
TNF-α	tumor necrosis factor-alpha
UV	ultraviolet radiation
VPA	valproic acid
H_2_O	water
